# Functional Characterization of Cnidarian HCN Channels Points to an Early Evolution of I_h_

**DOI:** 10.1371/journal.pone.0142730

**Published:** 2015-11-10

**Authors:** Emma C. Baker, Michael J. Layden, Damian B. van Rossum, Bishoy Kamel, Monica Medina, Eboni Simpson, Timothy Jegla

**Affiliations:** 1 Department of Biology, Penn State University, University Park, Pennsylvania, United States of America; 2 Department of Biological Sciences, Lehigh University, Bethlehem, Pennsylvania, United States of America; 3 Huck Institutes of the Life Sciences, University Park, Pennsylvania, United States of America; 4 Penn State University Graduate School, Summer Research Opportunities Program (SROP), University Park, Pennsylvania, United States of America; University of Waterloo, CANADA

## Abstract

HCN channels play a unique role in bilaterian physiology as the only hyperpolarization-gated cation channels. Their voltage-gating is regulated by cyclic nucleotides and phosphatidylinositol 4,5-bisphosphate (PIP_2_). Activation of HCN channels provides the depolarizing current in response to hyperpolarization that is critical for intrinsic rhythmicity in neurons and the sinoatrial node. Additionally, HCN channels regulate dendritic excitability in a wide variety of neurons. Little is known about the early functional evolution of HCN channels, but the presence of HCN sequences in basal metazoan phyla and choanoflagellates, a protozoan sister group to the metazoans, indicate that the gene family predates metazoan emergence. We functionally characterized two HCN channel orthologs from *Nematostella vectensis* (Cnidaria, Anthozoa) to determine which properties of HCN channels were established prior to the emergence of bilaterians. We find *Nematostella* HCN channels share all the major functional features of bilaterian HCNs, including reversed voltage-dependence, activation by cAMP and PIP_2_, and block by extracellular Cs^+^. Thus bilaterian-like HCN channels were already present in the common parahoxozoan ancestor of bilaterians and cnidarians, at a time when the functional diversity of voltage-gated K^+^ channels was rapidly expanding. NvHCN1 and NvHCN2 are expressed broadly in planulae and in both the endoderm and ectoderm of juvenile polyps.

## Introduction

Voltage-gated cation channels are fundamentally important for rapid transmembrane electrical signaling in neurons and muscle [1], and transcriptome analysis shows that the vast majority of mammalian orthologs are expressed in the brain [2]. However, these channels are widely distributed in eukaryotes and play fundamental signaling roles in all major eukaryotic clades that have been examined. Eukaryotic voltage-gated cation channels are highly diverse in terms of ion selectivity and gating mechanisms, but all can be traced back to a common origin in prokaryotes. They therefore form a single voltage-gated cation channel protein superfamily [1], comprising several structurally and functionally distinct gene families traceable to prokaryotes [1, 3–10]. This ancient set of voltage-gated cation channel gene families diversified greatly within the metazoan lineages. Exploring the functional evolution and origins of these metazoan gene families in detail has only recently become possible with the availability of diverse genomes and transcriptomes from basal metazoans and more diverse protozoans. For instance, voltage-gated K^+^ channels can be traced to prokaryotes, but the Shaker, KCNQ and Ether-a-go-go (EAG) gene families that encode our voltage-gated K^+^ channels appear metazoan specific [11, 12]. KCNQ channels and the functionally distinct Shab, Shal, Shaw, Eag, Elk and Erg subfamilies of the Shaker and Eag gene families emerged surprisingly late within the parahoxozoan lineage (cnidarians, placozoans and bilaterians) [11–13]. The distinct biophysical properties of Shaker, KCNQ and EAG family channels evolved in this parahoxozoan ancestor prior to the divergence of cnidarians and bilaterians [11, 12, 14–18]. Similarly, high-voltage-activated calcium channels predate the Metazoa, but the diversification of this gene family into functionally distinct L-type and N/P/Q/R-type channels likely occurred within the Metazoa [19]. Voltage-gated sodium channels also underwent significant functional diversification and refinement of selectivity specifically within the Metazoa [20].

In this paper we explored the functional evolution of hyperpolarization-gated cation channels (HCN), which form one of three major metazoan branches of the CNBD cation channel family. The CNBD family originated in prokaryotes [7] and derives its name from the presence of a cytoplasmic cyclic nucleotide binding domain which attaches to the channel core at the activation gate via a C-linker domain [21]. The CNBD family is one of the most widespread voltage-gated cation channel families in eukaryotes, including plant and *Paramecium* K^+^ channels [22–25]. The CNBD regulates voltage-gating through interactions with the canonical channel core (a voltage-sensor domain, or VSD, plus a pore domain) [26–29], but the mechanisms are not yet fully understood. Interestingly, the role of cyclic nucleotides in gating varies considerably in the CNBD family: CNG channels require cyclic nucleotide binding to open and are virtually insensitive to voltage under physiological conditions [26] while HCN channels are gated primarily by hyperpolarization, and cyclic nucleotides enhance activation to varying degrees [28]. In contrast, EAG family channels are gated by depolarization; their CNBD self-ligands and gating is insensitive to cyclic nucleotides [30–33]; the domain is therefore often referred to as a cyclic nucleotide binding homology domain (CNBHD) [31]. Interestingly, voltage controls VSD movement with the same polarity in HCN and EAG channels, but opposite coupling to pore opening reverses the voltage-dependence of HCN channels to create hyperpolarization-dependent activation [34]. Phosphatidylinositides also regulate CNBD-dependent gating of CNG and HCN channels and promote HCN activation by depolarizing the voltage-activation range [27, 35–37].

HCN channels occupy a unique physiological niche due to their unusual combination of hyperpolarization-gating and poor selectivity between Na^+^ and K^+^. They underlie I_h_ (or I_f_) *in vivo*, a depolarizing inward current activated by hyperpolarization. The classic HCN channel role is the pacemaker of intrinsically rhythmic neurons and the cardiac pacemaker cells of the sinoatrial node [38–40]. In many intrinsically rhythmic excitable cells, the after-hyperpolarization of an action potential activates I_h_. This current then provides the depolarizing drive for the next action potential. Dynamic regulation of I_h_ in the sinoatrial node alters heart rate and depends on both cAMP and PIP_2_ signaling [41, 42]. It is also now recognized that HCN channels play a key role in regulating signal integration in dendrites [43–45].

Mammals have 4 HCN channel orthologs with diverse gating properties [46–48], and HCN channels encoding hyperpolarization-gated I_h_ currents have been characterized in tunicates [49], sea urchin [50], honey bee [51] and lobster [52]. Classical HCN channels are thus widespread in the Bilateria, although they appear to have been lost in nematode *C*. *elegans* [8]. An HCN channel is present in a choanoflagellate *Salpingoeca rosetta*, demonstrating that the gene family predates the emergence of metazoans [53], but it has not been functionally expressed. Thus functional evolution of HCN channels prior to the emergence of Bilateria has not been directly examined.

Here we functionally characterized cnidarian HCN channel orthologs. Cnidarians are a sister taxa to the bilaterians within the Parahoxozoa [13], and thus it is possible to use cnidarian HCNs to infer which functional properties were likely present in these channels prior to the emergence of bilaterians. We show that classic I_h_-encoding HCN channels are present in the sea anemone *Nematostella vectensis* (Cnidaria), and thus I_h_ was likely present in ancestral parahoxozoans. Molecularly-identifiable HCN channels can also be found in the major basal metazoan lineages (ctenophore and sponge), but their functional properties have yet to be determined.

## Materials and Methods

### Cloning and Molecular Biology

We used a BLAST [54] search strategy relying on various bilaterian HCN channels as queries to search for HCN orthologs in genomes and transcriptomes from cnidarians, ctenophores and sponge. We identified HCN channel coding predictions or transcripts with full coverage of conserved domains from the following species *Nematostella vectensis* (starlet sea anemone) [55], *Orbicella faveolata* (star coral), *Acropora millepora* (stony coral) [56], *Hydra vulgaris* (hydra), *Dryodora glandiformis* (ctenophore) [57] and *Corticium candelabrum* (sponge) [58]. In some cases, gene predictions were manually optimized based on BLAST homology. *Nematostella vectensis* HCN channels (NvHCN1 and NvHCN2) were cloned by standard RT-PCR techniques using total RNA from adult polyps and transferred to the pOX vector [15] for expression in *Xenopus* oocytes. Genbank accession numbers for NvHCN1 and NvHCN2 are KT580854 and KT580853, respectively. *Salpingoeca rosetta* HCN (SrHCN) was synthesized with a *Xenopus*-optimized codon bias, and a CiVSP expression plasmid [59] was obtained from Jianmin Cui, Department of Biomedical Engineering, Washington University, St. Louis. cRNAs were prepared from linear templates using the mMessage mMachine kit (Life Technologies, Carlsbad, CA) and cleaned by LiCl precipitation prior to injection. Full-length NvHCN2 did not grow efficiently in bacteria, so the gene was maintained in pOX in two overlapping halves; transcription templates were prepared by overlap PCR with a high fidelity polymerase.

### Phylogeny

Only HCN channels for which we could identify the entire transmembrane ion channel core and C-terminal CNBD were used for phylogenetic analysis. *Nematostella* and *Orbicella* EAG and CNG channels were used as outgroups; cloning and characterization of the *Nematostella* EAG channels has previously been described [12, 17]. *Nematostella* CNG channels and *Orbicella* EAG and CNG channels were identified and assembled from genome annotations using a BLAST search strategy with human or *Nematostella* orthologs as queries. Five genes in each species encoded channels with best matches to CNG family channels in reciprocal queries of human sequence databases. Amino acid alignments for the phylogeny were built using CLUSTALW as implemented in MEGA6 [60], and manually adjusted as necessary to reflect structural conservation. A Bayesian inference phylogeny was constructed with Mr. Bayes v3.2 [61] using a mixed model, two independent runs of four chains and 1,000,000 generations. Trees were sampled at 1,000 generation intervals and the first 25% were discarded (burn in phase).

### *Xenopus* oocyte electrophysiology

*Xenopus* ovaries were obtained from Xenopus I (Ann Arbor, MI) and mature oocytes were isolated and defolliculated with 1–2 mg/ml Type II Collagenase (Sigma Aldrich, St. Louis, MO) as previously described [62]. Oocytes were injected with 50 nl of solution containing 5–50 ng cRNA dissolved in nuclease-free water supplemented with a 1:20 dilution Superasin (Life Technologies, Carlsbad, CA). We used 10–20 ng cRNA for CiVSP experiments, which was effective for inhibiting PIP_2_-dependent KCNQ channels in previous studies [11]. Subsequent to injection, oocytes were incubated at 18°C in ND98 (98 mM NaCl, 2 mM KCl, 1 mM MgCl_2_, 1.8 mM CaCl_2_, 5 mM HEPES, 2.5 mM Na-pyruvate, 100 U/mL penicillin, 100 μg/mL streptomycin, pH 7.2) for 1–5 days before recording.

HCN current recordings were made from whole oocytes under constant perfusion using standard two-electrode voltage clamp (TEVC) techniques. Borosilicate glass electrodes were filled with 3 M KCl and typically had resistances of 0.5–1 MΩ; bath clamp circuitry was placed in 1 M NaCl and connected to the recording chamber with a 1 M NaCl 1% agarose bridge. The recording solution consisted of in mM (98 NaOH, 2 KOH, 2 KCl, 2 CaCl_2_, 1 Mg Cl_2_, 5 HEPES, pH 7.5 with methanesulfonic acid, making the major anion methanesulfonate). 8-Br-cAMP and CsCl were added at the specified concentrations to this base solution. Data were acquired using a CA1B amplifier (Dagan Instruments, Minneapolis, MN) in TEVC mode and the pClamp 10 acquisition suite (Molecular Devices, Sunnyvale, CA). Data were sampled at 10 kHz and low pass filtered at 2 kHz.

Conductance-voltage (GV) relationships were determined from isochronal tail currents recorded at -50 mV after 4 s hyperpolarizing steps. Data were fitted with a single Boltzmann in Origin 2015 (OriginLab, Northampton, MA) using the equation *f*(V) = (*I*_1_ − *I*_2_)/(1 + *e* ^(V − V50) / s^) + *I*_2_, where V_50_ represents is the half activation voltage, s is the slope factor, and *I*_1_ and *I*_2_ represent the lower and upper asymptotes. Data from individual oocytes were fit independently and normalized to facilitate averaging. The Boltzmann fits shown in figure panels display the arithmetic means of the V_50_ and s values from individual fits. Activation time course was complex, including sigmoidal delay and multiple exponentials. Therefore, we quantified activation time course simply as the time needed for 50% activation at -100 mV. Deactivation time course was similarly complex, so we quantified the time required for 75% deactivation at 10 mV following a 1 s step to -100 mV. For measurements of the effect of CiVSP on the NvHCN1 GV, hyperpolarizing pulses were preceded by a 2 s step to +60 mV followed by a 400 ms step to the holding potential of -30 mV. In principle this -30 mV step will allow some recovery of PIP_2_, but we wanted to temporally isolate the hyperpolarizing activation steps from the +60 mV pulse to avoid direct PIP_2_-independent effects of holding voltage on the activation rate. For deactivation measurements in the presence of CiVSP, the +60 mV pulse was shortened to 1 s.

### *In Situ* Hybridization

*NvHCN1* and *NvHCN2* probes were cloned into pGEM-T (Promega, Madison, WI) using standard PCR and ligation protocols. DIG-labeled UTP mRNA anti-sense probes were generated as previously described (Wolenski et al., Nature Protocols 2013). *Nematostella* embryos were grown at 25°C in 1/3X Instant Ocean (Instant Ocean, Blacksburg, VA) artificial sea water and fixed at 12 hours post fertilization (hpf) (early gastrula), 24 hpf (late gastrula), 48 hpf (planula), 96 hpf (late planula), and 192 hpf (polyp) stages. mRNA *in situ* hybridizations were carried out using previously described methods (Wolenski et al., Nature Protocols 2013). Animals were imaged using a Nikon Eclipse Ni-E (Nikon, Tokyo, Japan) in conjunction with a Nikon DS-Ri2 color camera and Nikon NIS Elements software.

### Ethics Statement

*Xenopus* oocyte tissue samples were obtained from a licensed aquaculture vendor (Xenopus I). *Nematostella* tissue samples for RNA isolation were obtained from animals maintained according to best practices developed in the *Nematostella* community to optimize animal health.

## Results

**Fig 1:**
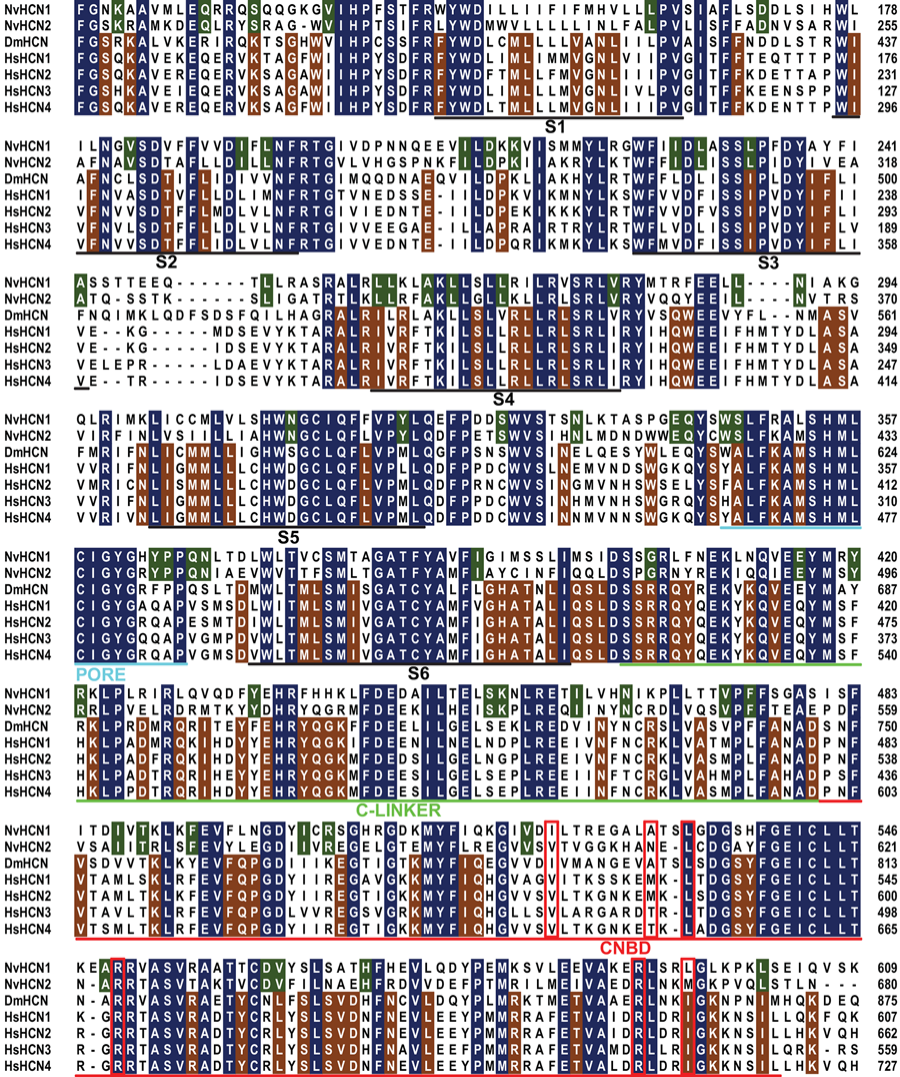
Multiple amino acid sequence alignment comparing sea anemone, human and *Drosophila* HCN channels. The alignment compares two putative HCN channels from the sea anemone *Nematostella vectensis* (NvHCN1 and NvHCN2) to HCN channels from *Drosophila* (DmHCN) and human (HsHCN1-4); residue positions are given at the right margin. Positions identical in all sequences are shaded in blue, and additional residues identical in *Nematostella* or the bilaterian sequences are shaded in green or brown, respectively. Approximate positions of 6 transmembrane domains (S1-S6) and the selectivity filter (PORE) are underlined and labeled in black or cyan, respectively. The C-linker is underlined in light green and the CNBD is underlined in red. Residues that contact cAMP in an HCN2 structure are boxed with a red outline. Accession numbers and full sequences are provided in S1 Table.

In order to determine if HCN channels with classical I_h_ properties evolved prior to the emergence and radiation of the bilaterians, we set out to examine the sequence and function of cnidarian HCN channels. We identified two putative HCN orthologs in BLAST searches of a cnidarian, the sea anemone *Nematostella vectensis*, and cloned full length coding sequences by RT-PCR. Fig 1 shows a sequence alignment spanning the region of conservation between the two *Nematostella vectensis* HCN channels, NvHCN1 and NvHCN2, and HCN channels from human and *Drosophila*. The alignment was generated using COBALT [63] and manually adjusted in the S4 region. Conservation is present through the transmembrane core of the channel (S1-S6) and the CNBD. 169/504 positions in the displayed alignment are identical in all sequences, with an additional 57 and 106 identical positions shared within either the *Nematostella* or the bilaterian sequences, respectively.

**Fig 2:**
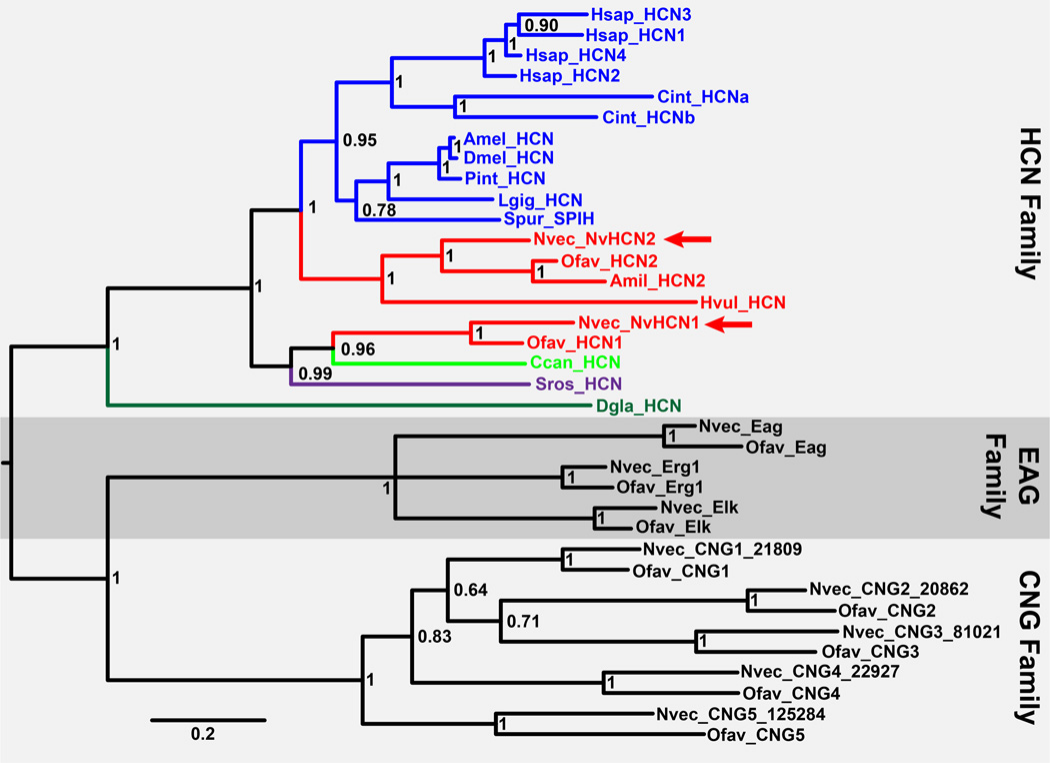
Bayesian inference phylogeny of the HCN channel family. Channels in the HCN family are color coded by phylogenetic group: bilaterians (blue), cnidarians (red), sponge (light green), ctenophore (dark green) and choanoflagellate (purple). The HCN family and the EAG and CNG family outgroups are indicated with shading and labels at the right margin. All outgroup sequences are cnidarian, but are not colored. Posterior probabilities for nodes are indicated and the scale bar is in substitutions/site. Channel names are given at branch tips with species prefixes as follows:; Amil, *Acropora millepora*, stony coral; Amel, *Apis mellifera*, honey bee; Ccan, *Corticium candelabrum*, sponge; Cint, *Ciona intestinalis*, tunicate; Dmel, *Drosophila melanogaster*, fruit fly; Dgla, *Dryodora glandiformis*, ctenophore; Hsap, *Homo sapiens*, human; Hvul, *Hydra vulgaris*, hydra; Lgig, *Lottia gigantea*, limpet; Nvec, *Nematostella vectensis*, sea anemone; Ofav, *Orbicella faveolata*, coral; Pint, *Panulirus interruptus*, lobster; Spur, *Stronglyocentrotus purpuratus*, sea urchin; and Sros, *Salpingoeca rosetta*, choanoflagellate. Full and aligned sequences for all branches are given in S1 Table.

While the sequence alignment presented in Fig 1 strongly suggests that NvHCN1 and NvHCN2 are true HCN orthologs, we performed a phylogenetic analysis to confirm this classification and examine the evolution of the HCN family within the metazoan lineage. Fig 2 shows a Bayesian inference phylogeny of the HCN channel family including NvHCN1, NvHCN2 and additional sequences we identified in BLAST searches from cnidarians (star coral, *Orbicella faveolata;* stony coral, *Acropora millepora;* hydra, *Hydra vulgaris*), ctenophore (*Dryodora glandiformis*) and sponge (Homoscleromorpha, *Corticium candelabrum*), compared to bilaterian and choanoflagellate HCNs. We used cnidarian EAG and CNG channels as CNBD channel family outgroups, and all putative HCN channel genes identified by BLAST grouped exclusively within the HCN family. However, the phylogeny does not resolve the relationship between the HCN family and the two outgroups. *Nematostella* and *Orbicella* share two HCN orthologs (NvHCN1 and NvHCN2 in *Nematostella*), suggesting a gene duplication occurring prior to the divergence of sea anemones and corals. *Acropora* appears to have orthologs of both genes, but only the NvHCN2 ortholog was analyzed here; the presence of NvHCN1 in *Acropora* is suggested by a small transcript fragment with 77% identity to amino acids 541–604 of NvHCN1 (EZ005349.1). NvHCN1 and NvHCN2 are only 48% identical over the same region (Fig 1). NvHCN1 groups with choanoflagellate and sponge HCN channels, while NvHCN2 groups with the bilaterian HCN channels. While this positioning could be interpreted to mean there were two ancestral HCN orthologs in a choanoflagellate/metazoan ancestor, with loss of one ancestor in all lineages except cnidarians, we prefer the interpretation that NvHCN1 and NvHCN2 were produced by a duplication within cnidarians prior to the divergence of the anthozoans lineages represented here. We have frequently observed asymmetric divergence in one or more paralogs of ion channel gene expansions restricted to cnidarians or bilaterians that causes separation of the paralogs within the tree [8, 16, 17]. More in-depth coverage of basal metazoan and cnidarian species could resolve this question.

The basal position of the *Dryodora* HCN sequence within the HCN family does not strictly follow species phylogeny (as it is basal to the *Salpingoeca* HCN channel), but is in keeping with genome analyses that show a high degree of sequence divergence in ctenophores and place them at the base of the metazoan phylogeny [57, 64, 65]. Discordance between species phylogenies and individual gene family phylogenies is a long-recognized and common issue in phylogenetics and is especially common for deeply diverging lineages [66, 67]. Another example of discordance observed here is the grouping of the sea urchin HCN SpIH with the protostome rather than deuterostome sequences. HCN-like sequences were widely present in cnidarian and ctenophore genomes and transcriptomes [57, 65, 68, 69], but we were only able to identify sequences sufficiently complete for robust phylogenetic analysis in *Nematostella*, *Acropora*, *Hydra*, *Orbicella* and *Dryodora*. In contrast, *Corticium* was the only one of 8 sponge transcriptomes [58] and 1 sponge genome [70] in which we identified an HCN channel. Previously, the Shaker K^+^ channel family was found exclusively within *Corticium* among these sponges [11, 58]. The *Corticium* transcriptome is rich in gene diversity compared to sponges from the family Silicea [58], but it remains possible that the limited spread of HCN channels in sponge is in part due to incomplete genome coverage. Similarly, HCN channels were not found in the genome of a second choanoflagellate *Monosiga brevicollis* [53, 71] and we did not find HCN channels in the placozoan *Trichoplax adhaerens* [72]. HCN channels are therefore widespread though not ubiquitous in parahoxozoans, the consensus basal metazoan lineages ctenophore and sponge [57, 65] and choanoflagellates. Further sequencing will be needed to determine whether the absence of HCN channels in some species is truly due to gene loss.

**Fig 3:**
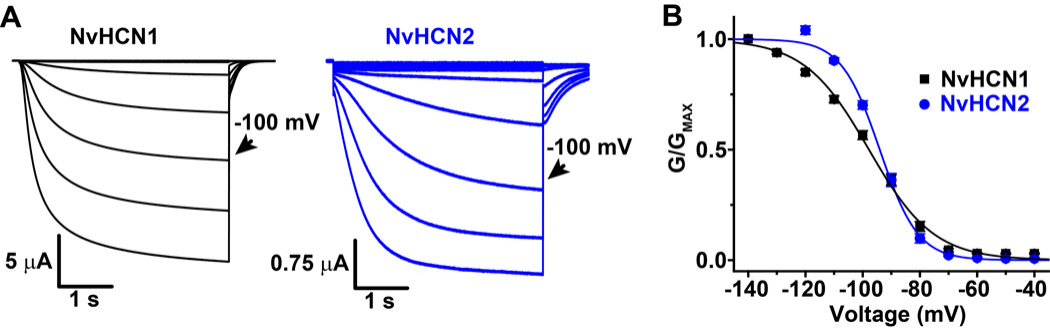
*Nematostella* HCN orthologs NvHCN1 and NvHCN2 are activated by hyperpolarization. (A) Families of inward currents recorded from *Xenopus* oocytes expressing NvHCN1 (left) and NvHCN2 (right) in response to 4 s hyperpolarizing voltage steps ranging from -40 to -120 mV in 10 mV increments. The holding potential was -30 mV and tail currents were recorded at -50 mV. Scale bars indicate time and current size and the -100 mV sweep is indicated with an arrow. (B) GV curves for NvHCN1 and NvHCN2. Fractional open probability (G/G_MAX_) was measured from isochronal tail currents recorded at -50 mV after 4 s hyperpolarizing steps to the indicated voltages as shown in (A). Data are normalized and show mean ± S.E.M. of measurements from individual cells. The smooth curves show single Boltzmann fits. V_50_, slope values and sample numbers are reported in Table 1.

**Fig 4:**
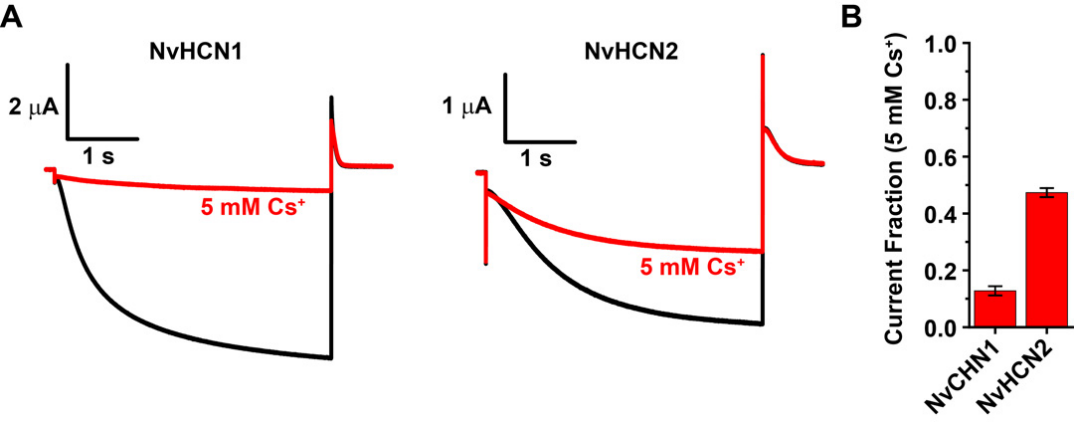
Cs^+^ block of NvHCN1 and NvHCN2. (A) Current traces recorded at -100 mV from oocytes expressing NvHCN1 or NvHCN2 before (black) and after (red) application of 5 mM Cs^+^ to the bath. The holding potential was -30 mV and outward tail currents were recorded at 0 mV. (B) Fraction of current remaining after application of 5 mM Cs^+^ for NvHCN1 and NvHCN2. Data show mean ± S.E.M. (n = 6 and 7 for NvHCN1 and NvHCN2, respectively).

We next tried to express the *Salpingoeca* (SrHCN) and *Nematostella* (NvHCN1 and NvHCN2) orthologs in *Xenopus* oocytes for electrophysiological characterization in order to better understand how the functional properties of the HCN family evolved. We were unable to obtain evidence for functional expression of SrHCN as measured by voltage clamp despite codon optimization, co-expression with mouse HCN orthologs or application of the cell-permeable cAMP analog 8-Br-cAMP. We therefore were unable to shed light on the functional phenotype of HCN channels prior to the emergence of the Metazoa. However, NvHCN1 and NvHCN2 expression yielded robust inward currents in response to hyperpolarization with typical features of bilaterian I_h_ (Fig 3A). NvHCN1 and NvHCN2 currents activate slowly in response to hyperpolarizing voltage steps and had very hyperpolarized voltage-activation (GV) curves with midpoints of -97.8 ± 1.1 mV and -94.0 ± 0.7 mV, respectively (Fig 3A and 3B, Table 1). Compared to mammalian HCN channels, these properties are most similar HCN4, which has the unusually slow activation gating and a hyperpolarized GV [48, 73]. NvHCN1 and NvHCN2 were also blocked by extracellular Cs^+^, a characteristic blocker of I_h_ (Fig 4A and 4B). At -100 mV, the NvHCN1 and NvHCN2 currents were blocked ~90% and ~50% by 5 mM Cs^+^, respectively (Fig 4B). We did not characterize the selectivity of the *Nematostella* HCN channel in detail, but tail currents reversed near -30 mV, which would require permeability to both Na^+^ and K^+^ in our recording solutions.

**Table 1:** Boltzmann fit parameters for NvHCN1 and NvHCN2 GV curves.

	Parameters	Mean	S.E.M.	n^a^
**NvHCN1**	**V**_**50**_ ^b^	-97.8	1.1	9
	**s** ^c^	11.3	0.3	
**NvHCN1 + 8-Br-cAMP** ^d^	**V**_**50**_	-97.8	1.5	7
	**s**	11.9	0.3	
**NvHCN1 + CiVSP**	**V**_**50**_	-103.7	1.4	7
	**s**	8.7	0.3	
**NvHCN2**	**V**_**50**_	-94.0	0.7	8
	**s**	6.7	0.4	
**NvHCN2 + 8-Br-cAMP**	**V**_**50**_	-87.9	1.5	8
	**s**	6.8	0.2	

^a^n, number of measurements

^b^V_50_, half-maximal activation voltage, mV

^c^s, slope factor, mV

^d^Bath applied at 2 mM

**Fig 5:**
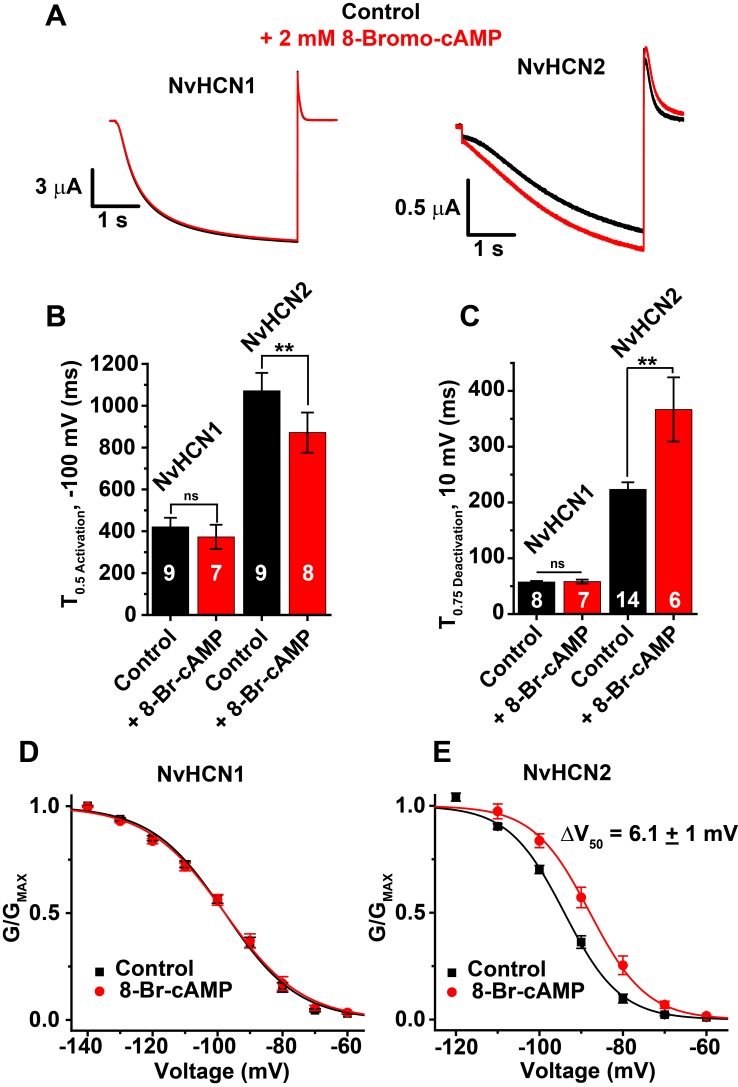
8-Br-cAMP enhances activation of NvHCN2 but not NvHCN1. (A) Examples of currents recorded in response to 4 s -100 mV voltage steps before (black) and after (red) bath application of 2 mM 8-Br-cAMP (-30 mV holding potential, tails recorded at 0 mV). (B) Half activation times at -100 mV for NvHCN1 and NvHCN2 before (black) and after (red) the addition of 2 mM 8-Br-cAMP. (C) 75% deactivation time at 10 mV for NvHCN1 and NvHCN2 with (red) and without (black) 2 mM 8-Br-cAMP. (D,E) GV curves for NvHCN1 and NvHCN2 with (red) and without (black) 2 mM 8-Br-cAMP. All data show mean ± S.E.M. and smooth curves in D and E show single Boltzmann fits of the data (parameters and sample number reported in Table 1). Sample numbers for (C) and (D) are shown on data bars. **Significance at p < 0.01 (t-test); ns, no significant difference.

We next bath applied 2 mM of the cell-permeable cAMP analog 8-Br-cAMP to voltage-clamped oocytes in order to determine whether NvHCN1 and NvHCN2 can be activated by cyclic nucleotides. 8-Br-cAMP accelerated voltage-activation and slowed deactivation of NvHCN2 but had no effect on the activation and deactivation kinetics of NvHCN1 (Fig 5A–5C). 2 mM 8-Br-cAMP significantly depolarized the GV curve of NvHCN2 by 6.1 ± 1 mV (p < 0.01, t-test), but did not alter the GV curve of NvHCN1 (Fig 5D and 5E, Table 1). In terms of cAMP sensitivity, NvHCN1 and NvHCN2 are most comparable to mammalian HCN3 which is completely insensitive to cAMP [74, 75] and mammalian HCN1 which has modest cAMP sensitivity with a GV shift of less than 10 mV [28]. However, a more direct comparison of cAMP sensitivity would require direct application to the intracellular face of excised patches, which we did not attempt here due to channel expression levels.

**Fig 6:**
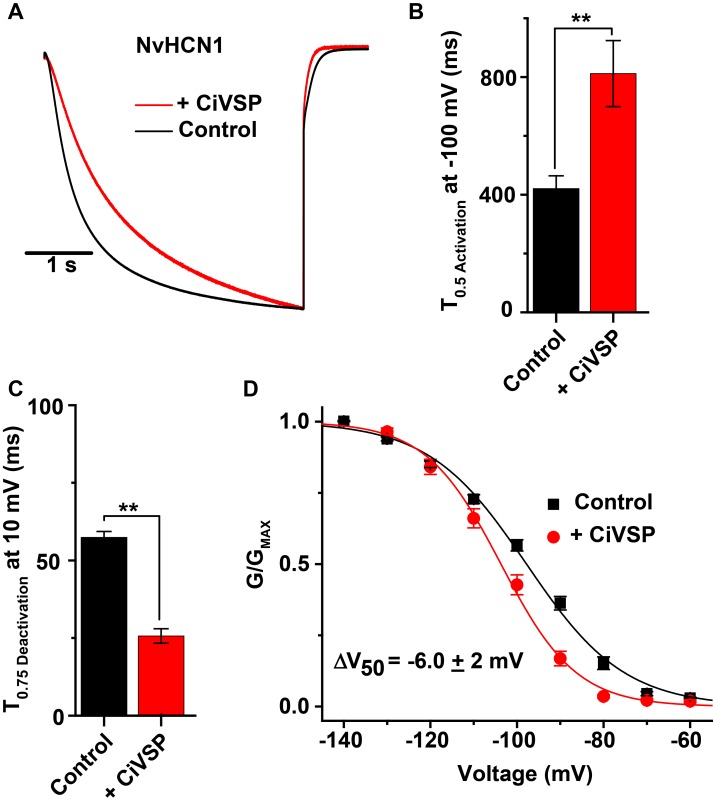
PIP_2_ depletion inhibits activation of NvHCN1. (A) Normalized current traces recorded in response to a 4 s -100 mV voltage step for control (black) and with PIP_2_ depletion by CiVSP (red). The holding voltage was -30 mV and tails were recorded at -50 mV. CiVSP was activated with a 2 s step to + 60 mV 400 ms prior to the hyperpolarizing current step. (B) Half activation time at -100 mV for NvHCN1 control (black) and for CiVSP-dependent PIP_2_ depletion (red). (C) 75% deactivation time at +10 mV for NvHCN1 controls (black) and for PIP_2_ depletion (red). (D) GV curves for NvHCN1 controls and NvHCN1 + CiVSP-dependent PIP_2_ depletion. All data show mean ± S.E.M.; ** in B and C indicates significant difference (p < 0.01, t-test, n = 7–9). Smooth curves in D show single Boltzmann fits of the data; fit parameters and sample numbers are reported in Table 1.

To determine whether Cnidarian HCN channels were sensitive to PIP_2_, we co-expressed NvHCN1 with a voltage-dependent phosphatidylinositol phosphatase from *Ciona intestinalis*, CiVSP, which depletes PIP_2_ in response to depolarization [76, 77]. We have previously observed strong depletion of PIP_2_ using CiVSP in whole oocytes as measured by inhibition of KCNQ channels which require PIP_2_ to open [11]. Activation of CiVSP with a depolarizing pre-pulse to +60 mV for 1 s significantly slowed the activation time course and sped the deactivation time course of NvHCN1 currents (Fig 6A–6C). These kinetic changes were reflected in a -6.0 ± 2 mV shift in the GV curve (Fig 6D, Table 1) when CiVSP was active. These results suggest that PIP_2_ enhances voltage-activation of NvHCN1, as has been observed for bilaterian HCN channels [27, 37, 41]. We compared the effects of CiVSP activation to controls recorded in the absence of CiVSP co-expression, because we assumed the chronically-depolarized resting potential of NvHCN1-expressing oocytes would lead to a baseline reduction in PIP_2_ in NvHCN1 + CiVSP oocytes. We did not examine the effect of CiVSP on NvHCN2 because it was difficult to simultaneously inject oocytes with sufficient amounts of both cRNAs, given the low expression level of NvHCN2.

**Fig 7:**
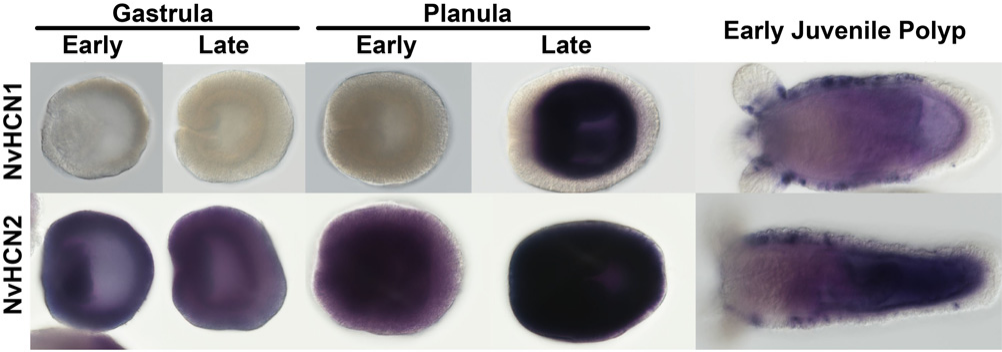
*In situ* hybridization of NvHCN1 and NvHCN2 expression. Examples of typical expression patterns in gastrulae, planulae and juvenile polyps are shown for *NvHCN1* (top row) and *NvHCN2* (bottom row). Animals were hybridized with anti-sense probes and detected colorimetrically (purple).

We examined the expression pattern of *NvHCN1* and *NvHCN2* in gastrula, planula and juvenile polyps using *in situ* hybridization (Fig 7). *NvHCN1* expression began in the late planula with strong, diffuse endodermal staining. Endodermal expression was maintained into juvenile polyps. *NvHCN1* expression was also detected in a punctate pattern in the body column and base of the tentacles in the juvenile polyp ectoderm. *NvHCN2* expression began as diffuse ubiquitous expression in the early gastrula, which increased to strong ubiquitous expression by planula stages. However, by juvenile polyp stages the ectodermal *NvHCN2* expression resolved into a punctate pattern while the endodermal expression retained a diffuse ubiquitous expression. The *NvHCN2* and *NvHCN1* expression patterns at polyp stages are remarkably similar, raising the possibility that heteromeric NvHCN channels could potentially form *in vivo* in *Nematostella*. The strength and diffuse nature of NvHCN1 expression at early developmental stages made it difficult to observe details of the expression pattern. We do not know the identity of the cells giving rise to the ectodermal punctae in juvenile polyps, but two cell types that commonly lead to punctate ectodermal labeling are neurons and cnidocytes [78, 79], which likely share a neuronal lineage [80].

## Discussion

We show here that I_h_-encoding HCN channels were present prior to the divergence of cnidarians and bilaterians. These findings add to a growing body of evidence that the major evolutionary innovations in metazoan voltage-gated cation channel signaling were complete by this time. Cnidarians and bilaterians also share 8 functionally conserved lineages of voltage-gated K^+^ channels [11, 12, 14–18] and voltage gated Na^+^ channels with strict or lax selectivity vs. Ca^2+^ [20]. All other functionally distinct molecular classes of the bilaterian voltage-gated cation channel superfamily can also be found in cnidarians [1, 8, 19, 81], but they have not yet been functionally expressed.

NvHCN1 and NvHCN2 collectively share several key functional features of bilaterian HCN channels beyond the classic hyperpolarization-dependent activation. These include block by Cs^+^, potentially activation by cyclic nucleotides for NvHCN2 and activation by PIP_2_ for NvHCN1 (deduced from inhibition following PIP_2_-depletion with CiVSP). The reason for the difference in cyclic nucleotide sensitivity between NvHCN1 and NvHCN2 is not clear because both have similar levels of conservation across 6 residues shown to contact cAMP in the structure of a mammalian HCN2 Channel (Fig 1, [21]), with 3 out of 6 residues identical and the others conservatively substituted. Mammalian HCN channels are identical at 5/6 of these positions yet vary considerably in cAMP sensitivity. Interestingly, all appear to bind cAMP, but differences in how the C-linker/CNBHD interacts with the channel core appear to determine the degree to which cAMP binding leads to channel activation [28, 75]. Thus the molecular basis for differential cyclic nucleotide sensitivity in NvHCN1 and NvHCN2 could lie outside the binding region. 8-Br-cAMP has been used extensively to explore the cAMP sensitivity of HCN channels [49, 52, 82] and typically shows close correspondence with results obtained by direct application of cyclic nucleotides to the intracellular face of excised patches [75, 83, 84]. Nevertheless, it remains possible the cAMP modulation we observed for NvHCN2 is indirect because we tested cyclic nucleotide sensitivity by bath application of 8-Br-cAMP.

The residues responsible for PIP_2_-dependent enhancement of voltage-activation in HCN channels have not yet been identified, but are believed to lie within the channel core [27]. Basic residues in the S4-S5 linker and S6 activation gate region interact with PIP_2_ in Shaker and KCNQ channels to influence voltage-gating [85, 86] and it is possible that a similar interaction takes place in HCN channels. HCN channels have basic residues within the same regions (see Fig 1), but their positions are not precisely conserved and their roles in PIP_2_ modulation have not been tested. PIP_2_ often modulates voltage-gated channels, including the sea urchin HCN channel SpIH, in a bimodal manner with opposing effects on voltage-gating and maximal current [27, 85, 87]. For instance, two basic residues in the C-linker are needed for a small PIP_2_-dependent inhibition of the maximal SpIH current [27]. Our CiVSP experiments did not have the resolution required to detect small changes in current size, and the presence of bimodal PIP_2_-modulation has not been confirmed in other HCN channels. We did not examine effects of PIP_2_ on current size further because neither basic residue is conserved in NvHCN1.

HCN channels are present in ctenophores, sponge and choanoflagellates, but we were unable to determine the functional properties of channels from these species in this study. Assuming that the I_h_ current phenotype tracks with the appearance of the HCN family, then I_h_ likely evolved in protozoans and was only later adapted for control of rhythmic excitability of neurons and muscle. It has been proposed that an I_h_-like current could have utility in regulating flagellum-based movements in choanoflagellates, similar to the role of I_h_ in regulating animal sperm motility [50, 53]. CNBD family channels found in other protozoans and prokaryotes so far do not belong to the HCN family [7, 23], suggesting that HCN might have arisen in a recent common ancestor of choanoflagellates and metazoans.

CNG channels share a similar pre-metazoan origin; metazoan-like CNG channels can be found in the choanoflagellate *Monosiga brevicollis* [8, 53, 71]. We show in Fig 1 that multiple CNG family orthologs are present in cnidarians, but we did not explore their functional properties in this study. EAG channels in contrast have a later origin and appear to be metazoan-specific. They are present in basal metazoans (ctenophores) but only diversified into the functionally distinct Elk, Eag and Erg subfamilies later within the Parahoxozoa [12, 17]. Thus it may be depolarization-gating rather than hyperpolarization-gating that is a “reversed” gating feature within the metazoan CNBD channel family. The polarity and importance of voltage-gating indeed appears to have been highly plastic within the CNBD family throughout its evolutionary history. The prokaryotic CNBD channel expressed so far is only weakly voltage-dependent [7], while plant CNBD family K^+^ channels include both hyperpolarization- and depolarization-gated channels [88].

Our *in situ* analysis shows several distinct patterns for *NvHCN* expression. First, at polyp stages both *NvHCN1* and *NvHCN2* are expressed ubiquitously within the endoderm and in punctate patterns within the ectoderm, which might overlap. *NvHCN1* is expressed strongly in the endoderm at the planula larval stages, and *NvHCN2* is expressed ubiquitously throughout much of early development. The discrete puncta expression in the polyp ectoderm is reminiscent of neural expression patterns [78, 89]. However, without transgenic reporters or a detailed expression analysis with neuronal markers, it is not possible to determine if these cells are neurons, cnidocytes or a previously uncharacterized cell type. The ubiquitous endodermal expression in *Nematostella* polyps is intriguing, because the myoepithelial cells that control the rhythmic contraction of the body column [17] are present throughout the endodermal tissue [90]. It is not yet known if contractile rhythmicity in *Nematostella* is intrinsic to the myoepithelium or driven by neuronal input. Regardless, it would be interesting to determine if HCN channels contribute to rhythmic behaviors in *Nematostella*, which could point to an established role in rhythm generation in ancestral parahoxozoans prior to the divergence of cnidarians and bilaterians.

## Supporting Information

S1 Table Sequences used in Fig 1 phylogeny. (XLSX)
